# A Unique Acquired Athletic Dermatologic Condition in a Half Marathon Participant: An Autobiographical Case Report of Sports-Associated Clothing Related Axillary Tangled Clumped Hairs (SCRATCH)

**DOI:** 10.7759/cureus.85161

**Published:** 2025-05-31

**Authors:** Philip R Cohen

**Affiliations:** 1 Dermatology, University of California Davis Health System, Sacramento, USA; 2 Dermatology, Touro College of Osteopathic Medicine, Vallejo, USA; 3 Maples Center for Forensic Medicine, University of Florida College of Medicine, Gainesville, USA

**Keywords:** acquired, axilla, clothes, clumped, hair, running, scratch, sports, tangled, walking

## Abstract

Runners are susceptible to developing sports-related disorders. A 66-year-old man developed a unique cutaneous condition affecting his armpits while participating in a 13.1-mile race. Friction from the shirt he was wearing resulted in the development of sports-associated clothing related axillary tangled clumped hairs (SCRATCH). Removal of the hairs that had become tangled and clumped resulted in temporary localized patches of alopecia. The incidence of SCRATCH remains to be established. The condition results from the seams of the shirt rubbing against the axillary hairs and creating a mass of tangled and clumped hairs that are painful when the runner moves their arms. Removal of the hair masses can result in focal areas of alopecia in the affected axillae; subsequently, the tractional alopecia created by the hair removal resolves. SCRATCH does not occur in individuals who regularly shave their axillary hairs. The condition can be prevented by shaving or cutting the axillary hairs prior to running, possibly by applying a salve to the axillary hairs so that they do not adhere to the shirt, or by wearing shirts that do not rub against the axillae. A comprehensive summary of sports-associated dermatologic conditions in runners is presented. In conclusion, individuals who participate in ambulatory activities are susceptible to many dermatologic conditions related to their sport and SCRATCH can be added to the list of potential cutaneous disorders that can occur in runners.

## Introduction

Dermatology includes the study of not only skin and mucous membranes, but also hair and nails. Sports dermatology involves conditions that are associated with athletic activities that result in conditions that affect these sites [1-7]. Dermatological conditions that occur in athletic participants include environment-induced disorders, immunologic disorders and inflammatory conditions, infections, neoplasms, trauma-related conditions, and miscellaneous conditions; walking and running are both activities that can potentially result in a sports-related dermatosis [1-20].

Conditions affecting the hair can be endogenous or exogenous. Sports-related friction to the hair can alter its morphology and even result in alopecia [1,5]. Friction of the clothes that rub against the athlete’s intertriginous skin, such as the axillae, can result in tangled and clumped hair. This condition is referred to as sports-associated clothing related axillary tangled clumped hairs (SCRATCH).

SCRATCH is a unique cutaneous condition that affects the axillary hairs. SCRATCH can only occur in an individual who does not regularly shave their axillary hairs. The incidence of SCRATCH remains to be determined.

The treatment of SCRATCH involves detaching the tangled and clumped hairs from the body. Options for preventing the condition include shaving or cutting the axillary hairs prior to walking or running races and wearing shirts that do not rub against the axillae. Applying a salve to keep the axillary hair adherent to the body is also postulated to prevent SCRATCH.

A 66-year-old man is reported to have developed SCRATCH while participating in a half marathon. The affected areas were tender when he removed his shirt, and the condition resolved after removing the affected hair. However, the manual traction applied to remove the hair masses resulted in localized traction alopecia, which subsequently resolved spontaneously.

The cutaneous manifestations of SCRATCH are described including the clinical presentation, alteration to the affected hairs, and localized traction alopecia experienced by the patient from the removal of the tangled and clumped hairs. In addition, the pathogenesis, treatment, and measures to prevent the condition are discussed. Also, dermatologic conditions potentially associated with walking and running are summarized.

## Case presentation

A 66-year-old man participated in a half marathon on April 12, 2025, in San Diego, California. He had a history of atrial flutter-associated cerebrovascular accident, hyperlipidemia, hypertension, gout, and severe spinal stenosis with postoperative cerebrospinal fluid leak. He has mild androgenetic alopecia (stage II Norwood-Hamilton classification); none of his family members have developed tangled or clumped axillary hair.

The 13.1-mile race began at 6:30 am and the ambient temperature when the race began was 56 degrees Fahrenheit. He walked the course at a pace of 17 minutes per mile. The temperature had increased to 65 degrees Fahrenheit when he had completed the race after three hours and 42 minutes. 

After one hour of walking, he developed asymptomatic swelling of both hands. This was like the hand changes he had experienced during prior half marathons. Both of his hands and their digits became enlarged, and he could not make a fist.

During the final three miles (which nearly took one hour to complete), he felt the long-sleeved shirt that he was wearing painfully rubbing on the hairs of his axillae. On a scale from zero (no pain) to 10 (severe pain), the axillary pain he was experiencing was between a two to four depending on how vigorously he would move his arms; if he kept his arms at his side with minimal back and forth activity the pain level only zero to one. The locations of the tangled and clumped hairs transiently became painful when the skin was stretched while he removed his shirt after completing the race.

**Figure 1:**
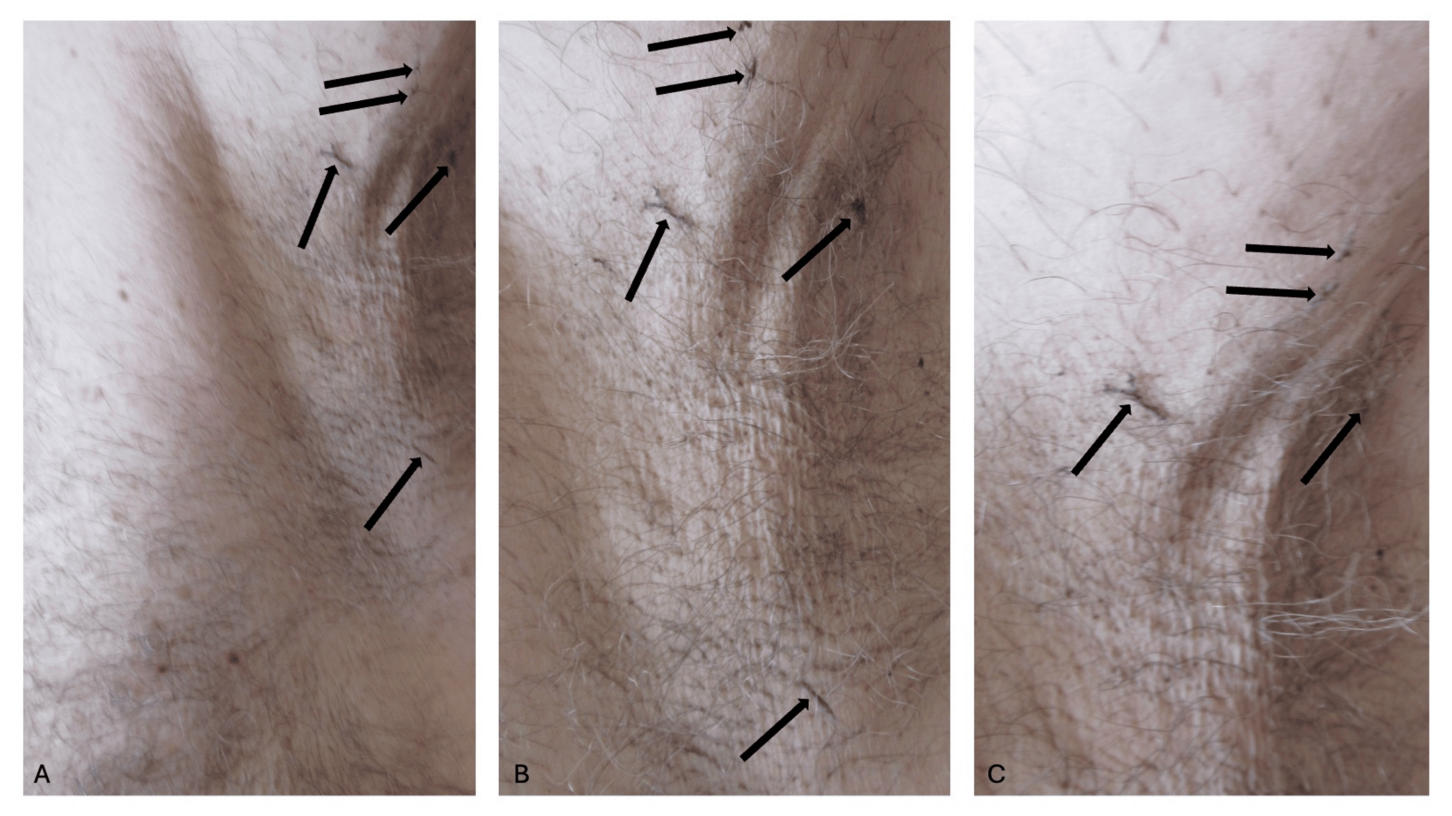
Sports-associated clothing related axillary tangled clumped hairs (SCRATCH) Distant (A) and closer (B and C) views of the left axilla of a 66-year-old man who completed a 13.1-mile race wearing a long-sleeved shirt. Some of the axillary hairs (black arrows) are either tangled in a linear morphology or clumped in a matted presentation.

Approximately three to five percent of the hairs in both of his axillas were tangled and clumped (Figure 1). Some of the hairs had become tangled in a linear distribution against the skin. Other hairs were clumped and had a matted appearance.

**Figure 2:**
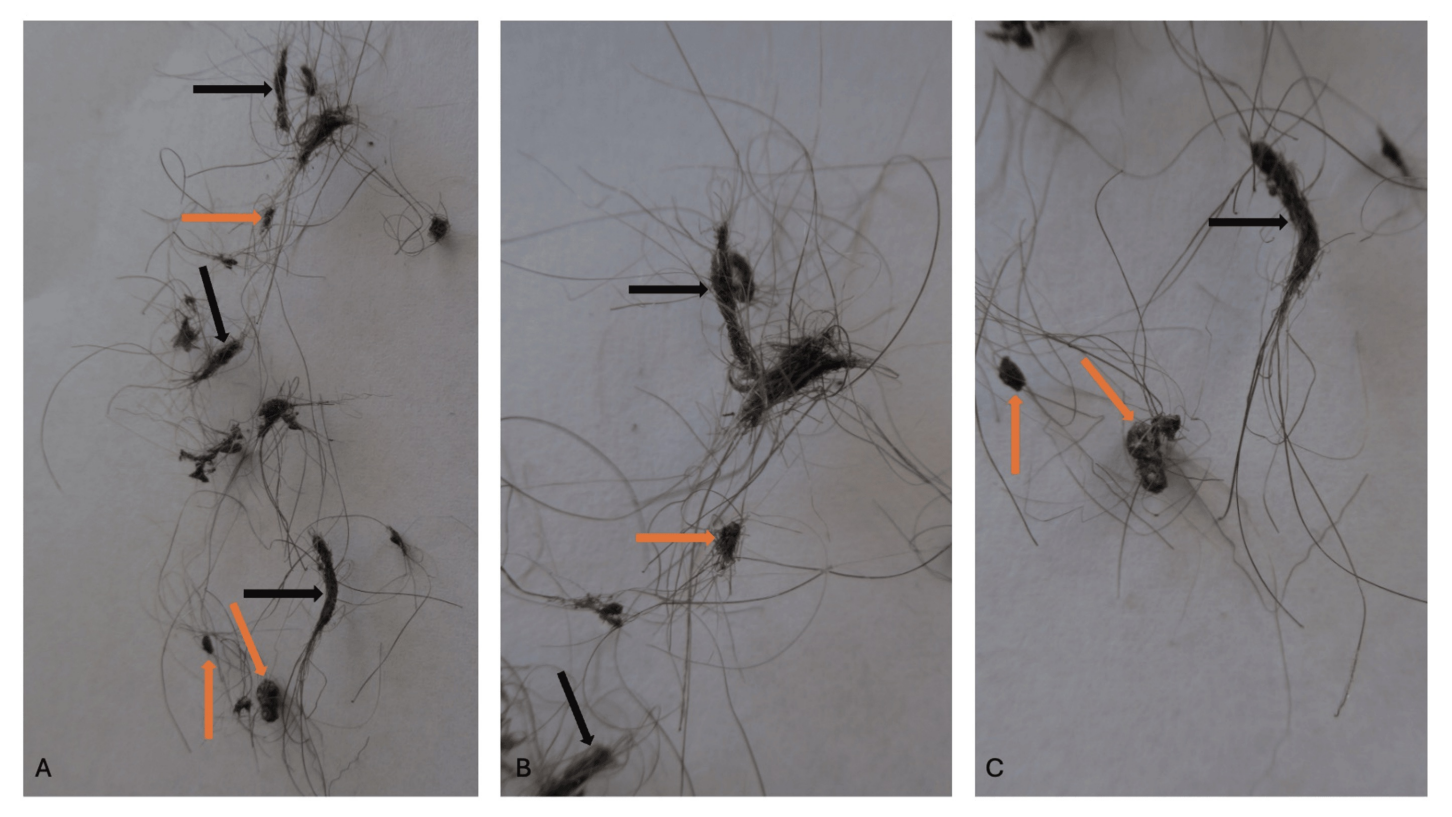
Tangled and clumped axillary hairs after removal Distant (A) and closer (B and C) views of the sports-associated clothing related axillary tangled clumped hairs (SCRATCH) following removal of the altered hairs. The clothing rubbed against the axillary hairs; however, threads from the shirt are not observed in either the linearly tangled hairs ranging from six to 12 millimeters in length (black arrows) or the clumped matted hairs ranging from two to five millimeters in diameter (orange arrows).

The tangled and clumped hairs were swiftly removed by pulling them from his axillas. Examination of the removed hairs showed linear arrangements of tangled hairs ranging from six to 12 millimeters in length. The examination also showed several hairs clumped together ranging from two to five millimeters in diameter (Figure 2). 

All the symptoms caused by the tangled and clumped hairs completely resolved after the affected hairs had been removed. This occurred about 90 minutes after he completed the race. Therefore, the total duration of the symptoms from the altered hair was about two and a half hours.

**Figure 3:**
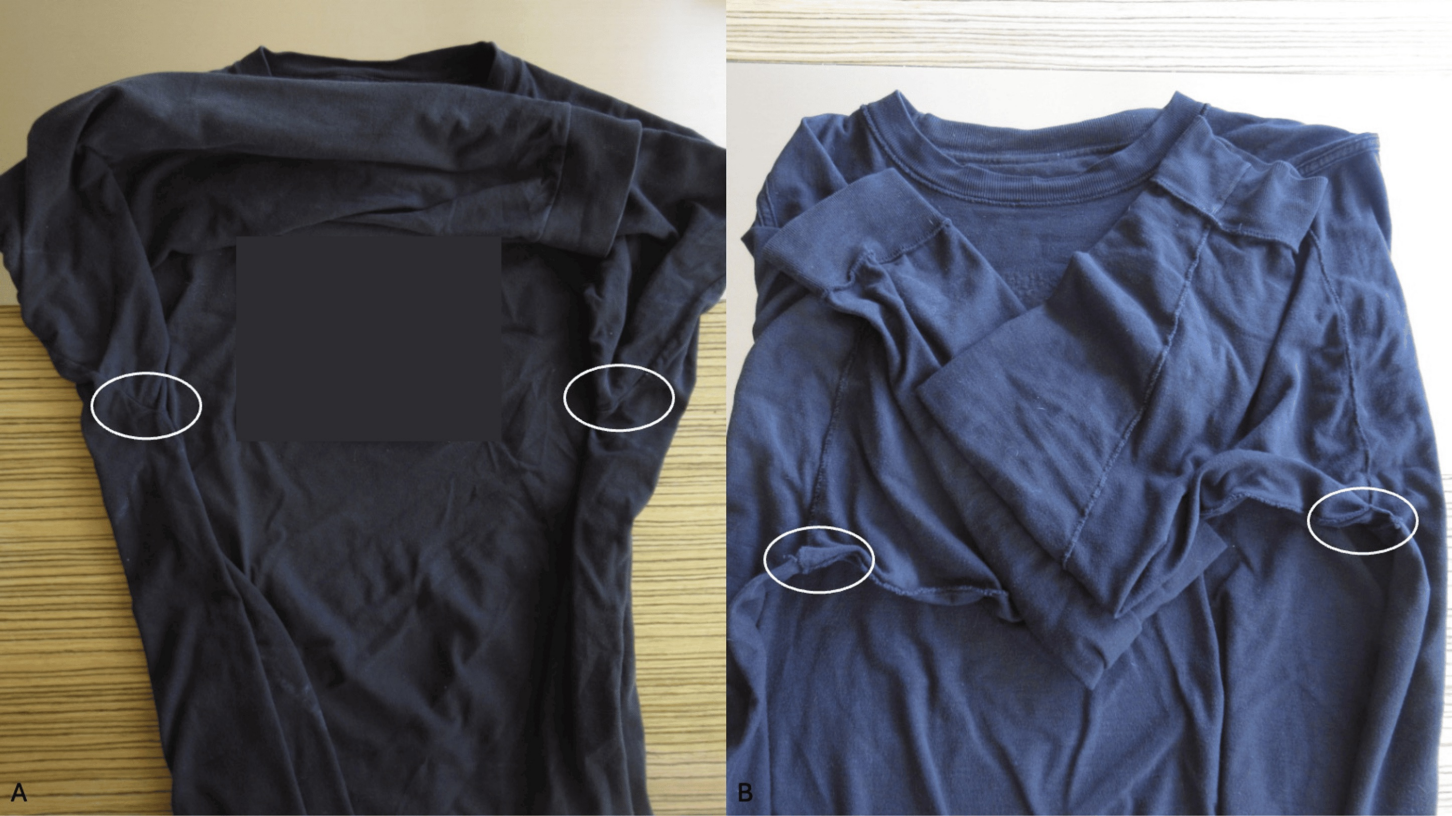
The long-sleeved shirt that the man was wearing during the race The external view (A) and the internal view (B) of the blue, 100 percent cotton, shirt that the man was wearing during the half marathon; the white ovals surround the portions of the shirt that rubbed against the axillae. On the internal view (B) of the shirt, frayed strands of material extending from the seams (within the white ovals), can be seen in the axillary regions.

The long-sleeved blue shirt he had been wearing was a 100 percent cotton (Figure 3). There were no tears or holes in the axilla. However, a view of the inside surface of the shirt showed the material used to sew the seams of the shirt was focally thickened and extended from the seam; no detached hairs were observed in the seams of the shirt.

**Figure 4:**
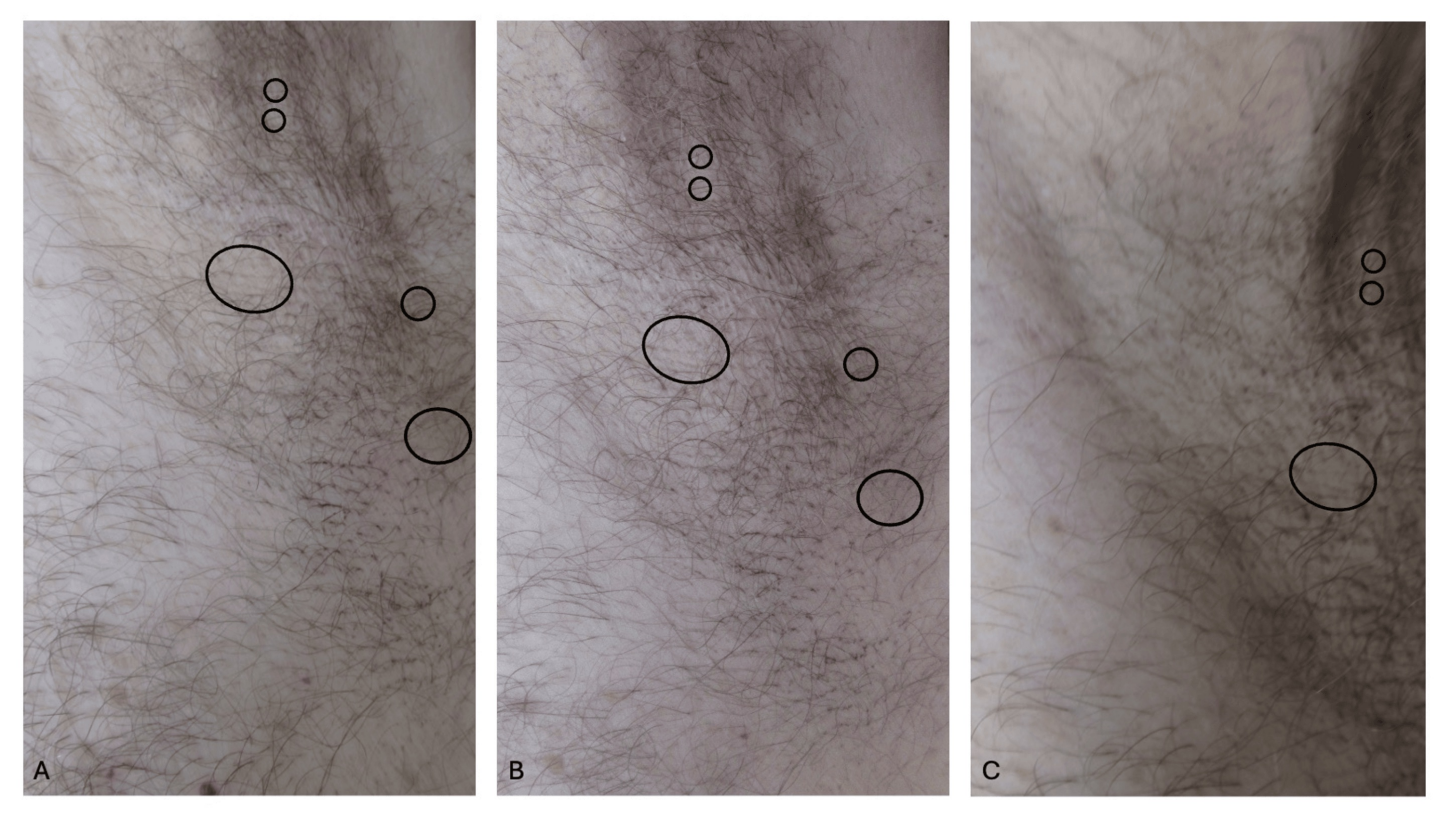
Focal patches of traction alopecia that resulted from the removal of the sports-associated clothing related axillary tangled clumped hairs (SCRATCH) Distant (A) and closer (B and C) views of the left axilla after removal of SCRATCH. There are focal patches of alopecia (areas within the black circles), where the altered hairs had been located, after the brisk removal of the tangled and clumped axillary hairs by pulling them from the skin.

Examination of the axillas after removal of the hairs demonstrated focal areas of alopecia (Figure 4); additional assessment and investigation (such as dermoscopy, trichogram, or biopsy) were not performed. Follow-up examination one month later showed early regrowth of hairs that had been removed. The post ambulatory swollen hands (POTASH) spontaneously resolved; the hands and their digits returned to their normal thickness (and he was able to make a tight fist) within two hours after he had completed the race.

## Discussion

Sports dermatology is characterized by skin diseases that occur in athletes. Several skin manifestations of running have been observed [1-20]. They include environment-induced disorders (Table 1) [1-5,7].

**Table 1:** Environment-induced disorders associated with running

Disorder	Comment	References
Actinic damage	Many runners do not like to wear sunscreen since it drips from their forehead and irritates their eyes. These individuals may develop phototoxic reactions (sunburn), actinic keratoses, and skin cancers.	[2,7]
Cheilitis simplex	Dry, cracked, or fissured lips; there may be superficial peeling of the skin and mucosa. The condition can be precipitated by old and/or dry weather and exacerbated by lip licking, in which the saliva can be an irritant.	[4]
Cold panniculitis	Exposure to cold during running can cause inflammation of the subcutaneous fat.	[2]
Foreign body reaction to exogenous material or injury	Some runners have decided to run barefoot. While they are running on the roads or other surfaces, they risk injury from foreign material such as splinters, glass shards, and stones; they also may develop foreign body reactions if these materials penetrate the skin.	[2]
Frost bite	Blisters may develop within a day. Subsequently, the runner may experience burning and throbbing of the affected area that can last for several weeks.	[2,5]
Frost nip	Penile frost nip developed on a jogger who was only wearing polyester trousers and cotton underwear with an anterior opening during his running in subfreezing temperatures. Frostnip most frequently occurs on the skin over the cheeks, chin, ears, and nose; it is often induced by the combination of exercise, wind (such as a windchill factor), and cold weather (with temperatures below freezing).	[1,7]
Ingrown hairs	Friction and chronic rubbing can result in the distal hair shaft penetrating the epidermis. Intermittent shaving may predispose to this problem.	[4]
Insect bites and stings	In a study of 76 road runners, these occurred in six (7.9 percent) of the participants.	[4,7]
Miliaria	Running in hot and humid climates may create miliaria. Miliaria crystallina (sudamina) presents as tiny asymptomatic fluid-filled blisters when eccrine sweat accumulates beneath the stratum corneum. Miliaria rubra presents as tender or pruritic, deeper, erythematous lesions from rupture of the eccrine sweat glands.	[2,7]
Pernio	This is also referred to as chilblains. Small patches of inflamed skin present as itchy, red or blue bumps on the toes, fingers, nose and ears of individuals who run in the cold.	[2,7]
Polymorphous light eruption	Photosensitivity disorders in susceptible individuals were also observed on the arms and chests.	[3]

Immunologic and inflammatory disorders have also been noted in runners (Table 2) [1-7].

**Table 2:** Immunologic disorders and inflammatory conditions associated with running

Disorder	Comment	References
Acne mechanica	This can result from the combination of predisposing factors, which include friction, heat, and occlusion. It can present or flare on the foreheads of runners who wear headbands to keep hair and perspiration away from their eyes.	[5,7]
Acne vulgaris	Acne is an inflammatory condition of the skin that frequently worsens in locations of friction and sweat. It presents as inflammatory papules, pustules, nodules, and cysts.	[2,7]
Allergic contact dermatitis	Topical medicines (such as topical analgesics containing arnica, methyl salicylate, and trolamine salicylate or topical antibiotics such as bacitracin, mupirocin, neomycin, and polymyxin B) used for injuries and contact with components in the running shoes can cause dermatitis. The athletic tape or rubber-backed tape may contain formaldehyde resin. The rubber soles may contain ethyl butyl thiourea, dibenzothiazole disulfide, or mercaptobenzothiazole. The dermatitis presents as unusual geometric shapes or linear shapes; the well-defined erythematous lesions can be vesicles or crusted and/or eroded plaques or both. The dermatitis may also have been due to the chemical additives in the clothing the runners were wearing.	[1-3,5-7]
Capillaritis with purpuric lesions	In a study of runners of an ultramarathon (250 kilometers over six days), this was noted in some of the runners.	[3]
Conjunctivitis	In a study of 76 road runners, this occurred in two (2.6 percent) of the participants.	[4]
Dermatitis	Individuals with atopic dermatitis may note flares produced by not only friction, irritation, and sweating but also excessive hygienic practices. In a study of 76 road runners, non-specific eczema occurred in one (1.3 percent) of the participants. Irritation dermatitis can also be associated with the rubber components in the footwear of runners, especially in the setting of sweating.	[4,6,7]
Exercise-induced anaphylaxis	This can be life-threatening. At highest risk are individuals who are atopic with food allergies. It begins with pruritus, cutaneous warmth, and erythema; it progresses to urticaria and angioedema.	[1,2,5]
Physical urticaria	These included cholinergic urticaria, which presents as small well-defined erythematous wheals related to exercise, exertion, overheating of the body from the temperature of the environment, or stress; it occurs within two to 30 minutes after running has begun. The physical urticarias also include cold urticaria (wheals in areas of cold exposure and particularly cold-water contact), solar urticaria (itching, erythema, and burning wheals in sun-exposed areas), and vibratory urticaria and angioedema.	[1,2,5,6]
Plant dermatitis	In a study of 76 road runners, this occurred in four (5.3 percent) of the participants.	[4]

Several bacterial, fungal, and viral infections have been observed in runners (Table 3) [1-11].

**Table 3:** Infections associated with running

Condition	Comment	References
Erythrasma	This is caused by a bacterium, Corynebacterium minutissimum, which demonstrates a coral red color after illumination using a Wood lamp. It is usually asymptomatic and most commonly occurs in the groin, axillae, inframammary, and between the toes.	[2,6,8]
Folliculitis	Bacteria-associated follicular papules and pustules can develop in some runners. These are frequently caused by Staphylococcus aureus (either susceptible or resistant to methicillin). This may progress to deeper and more extensive infections such as furunculosis.	[2,5,6]
Gram-negative toe web infections	These bacterial infections can be caused by Pseudomonas aeruginosa (and present with foul-smelling, severely macerated, eroded, erythematous areas between the toes and extending to the soles of the feet.	[1]
Herpes virus infection	Herpes simplex virus infection may be triggered by sunlight or wind or cold; recurrences of prior herpes simplex infections may occur in runners.	[2,4]
Intertrigo	This is a yeast infection, most commonly caused by Candida albicans, that occurs under the breast and in other flexural areas such as the axilla, groin, and between the toes. It presents as tender macular erythema with scaling.	[2,3]
Methicillin-resistant Staphylococcus aureus infection	This antibiotic-resistant bacterial organism is frequently encountered in athletes. Indeed, athletes are a risk group for this variant of Staphylococcus infection. The lesions can present as folliculitis, cellulitis and/or tender erythematous abscesses or boils.	[2-6]
Methicillin-susceptible staphylococcal aureus infection	In a study of runners of an ultramarathon (250 kilometers over six days), staphylococcal skin infection was observed as a complication of foot injuries in some of the runners.	[3,5,6]
Molluscum contagiosum	This is a pox virus infection that presents as umbilicated papules. It is more common in combat sports participants, yet it has been observed on the legs of cross-country runners.	[9]
Onychomycosis	Fungal infection of the nail plate caused by either dermatophyte (usually Trichophyton rubrum or less commonly Trichophyton mentagrophytes and Epidermophyton floccosum), yeasts (Candida), or non-dermatophyte molds.	[2]
Pitted keratolysis	This is a superficial gram-positive bacterial infection caused by either Corynebacterium species, Kytococcus sedentarius, or Dematophilus congolensis. The infection occurs on the pressure-bearing areas of the soles of the feet, such as the heel or proximal to the toes and appears as asymptomatic discrete, circular, crater-like pits. Often there is an accompanying odor to the affected area. Hyperhidrosis and sports activities utilizing occlusive footwear are risk factors for this infection.	[5-7,10]
Pityrosporum folliculitis	This typically occurs on the upper trunk and proximal upper extremities as erythematous papules and pustules. It is more prevalent in the summer months and in the setting of heat, sweating and poor personal hygiene habits. Like tinea versicolor, this variant of folliculitis is caused by Malassezia furfura.	[5]
Plantar verruca	Warts are caused by human papillomavirus. They typically present on the soles of the foot as hyperkeratotic papules and plaques.	[1,2,5]
Streptococcal skin infections	In a study of runners of an ultramarathon (250 kilometers over six days), streptococcal skin infection was observed as a complication of foot injuries in some of the runners.	[3,5,6]
Tinea cruris	Superficial dermatophyte infection of the groin folds.	[2,6]
Tinea pedis	Dermatophyte infections can cause superficial fungal infections of the feet. The most common causes are Trichophyton rubrum (presenting as interdigital and scaly moccasin types) and Trichophyton mentagrophytes (presenting as inflammatory or vesicular variants). When the tinea pedis (athlete’s foot) infection is associated with Trichophyton rubrum, it has been referred to as runner’s rubrum.	[1-5]
Tinea versicolor	This is a superficial fungal infection that presents as either hyperpigmented, hypopigmented, or erythematous plaques with peripheral scaling. It is caused by the dimorphic, lipid-dependent, yeast from the genus Malassezia (furfura, globosa, or sympodialis); the genus was previously referred to as Pityrosporum.	[2]

Not only precancerous lesions (actinic keratoses), but also skin neoplasms (including basal cell carcinoma, squamous cell carcinoma, and malignant melanoma have developed in runners (Table 4) [1,2,4,5,11].

**Table 4:** Neoplasms associated with running

Neoplasm	Comment	References
Actinic keratoses	Often presenting as erythematous scaly plaques on sun-exposed areas of the body which microscopically demonstrate atypical keratinocytes restricted to the basal layers of the epidermis. These lesions have the potential to develop into skin cancer.	[4]
Basal cell carcinoma	Marathon runners have an increased risk for the development of basal cell carcinoma.	[1,2,4,5,11]
Malignant melanoma	Marathon runners have an increased risk for the development of malignant melanoma.	[1,2,5,11]
Squamous cell carcinoma	Marathon runners have an increased risk for the development of squamous cell carcinoma.	[1,2,5,11]

Numerous trauma-related conditions have occurred in athletes who run (Table 5) [1-7,12-15].

**Table 5:** Trauma-related conditions associated with running

Condition	Comment	References
Abrasions and cuts	In a study of 76 road runners, these occurred in five (6.5 percent) of the participants.	[4]
Alopecia walkmania	A woman who wore a tight-banded, wide-stripped, heavy headphone set during her daily jogging developed jogger’s alopecia (hair loss that was linear and transverse). The traction alopecia corresponded to the distribution of the headphones and resolved when she switched to a lighter headpiece.	[1,2,12]
Animal bites	In a study of 76 road runners, this occurred in one (1.3 percent) of the participants.	[4]
Athlete’s nodules	These are nodules or pseudonodules created by chronic pressure from wearing tight-fitting athletic shoes; they have been referred to as Nike nodules.	[2,5,6,13]
Blisters	Epidermal splits result from the running-related horizontal shearing forces; histologically, the splits occur in the mid to lower malpighian layer of the epidermis or through the lamina lucida at the junction of the epidermis and dermis. The most common locations of blisters are the heel (behind the calcaneus), under the metatarsals, and at the distal phalanges of the feet. Ill-fitting shoes (either too tight or too loose), heat, moisture, and overtraining are risk factors for blister development.	[1,2,4-6]
Calluses	Hyperkeratotic skin is often over bony prominences. In contrast to a corn, a callus does not have a central hyperkeratotic core. Unlike warts, they do not have capillary thrombi.	[2,4-7]
Chafing	This is common in runners; it presents as a superficial dermatitis that develops from skin surfaces that rub together. The skin is erythematous and painful. This can occur associated with waist packs, and around the perimeter of intertriginous areas.	[1,2,4]
Corns	Punctate papules with a deep hyperkeratotic core; they are typically over a bony prominence. Palpation of the lesion is usually associated with pain. They are typically located on the skin overlying the distal head of the metatarsals and the plantar surface of the great toes. These are either soft (when they occur between digits, white, and may become macerated) or hard (when they are plantar and cause pain following direct pressure or weight bearing). Unlike warts, they do not have capillary thrombi.	[2,5,6]
Erosions	These include ruptured blisters and trauma-related erosions from accidental contact of the skin with injury-producing superficial wounds and ulcers.	[3]
Frictional alopecia of the distal legs	This is a tractional alopecia localized to the lower extremity. It is asymptomatic and results from the repetitive rubbing of the person’s socks or footwear, or both against the hair-containing skin below the knee. A middle-aged man, who became a soccer referee, developed hair loss on both of his legs that corresponded in distribution to the tight-fitting socks his uniform required him to wear. that extended up his legs higher than his calves. He was constantly running up and down the soccer field, two to three nights each week. Within three months after the soccer season began, the asymptomatic traction alopecia on his legs developed.	[14]
Jogger’s nipples	This is a unique form of chafing injury that occurs between the runner’s shirt and their nipples. The repetitive friction of the runner’s shirt rubbing against the surface of the nipple causes the painful erythematous erosions on the nipples and the surrounding areola. Fissures and bleeding may also occur. This is not only observed in men who wear shirts made of coarse fibers, but also in women who run without bras. Factors that promote this condition are long runs (such as marathons) in cooler environments (when the nipples may be erect) and moist shirts.	[1,2,4-6]
Jogger’s toe	The nail plate of the second toe is most susceptible; however, the great toe and the periungual areas of the third to fifth toes are susceptible to traumatic injury. Subungual hematomas can occur acutely, and subungual hyperkeratosis can occur after chronic repetitive contact of the distal toe with the toe box of the sneaker. In addition to subungual hematoma or subungual hyperkeratosis, other dystrophy of the toenails may develop nail plate opacification, nail plate thickening, onychocryptosis (ingrown toenails), onychomadesis (proximal nail plate shedding), and onycholysis (distal nail plate shedding).	[1-6,15]
Piezogenic pedal papules	The papules are subcutaneous fat that has herniated through the collagen matrix of the reticular dermis. The lesions can either be asymptomatic or painful. They present as yellow to white protuberances on the medial or posterolateral areas of the heel. Elevation of the feet for a few minutes will result in the disappearance of the symptoms and papules; however, the papules reappear when the person stands. A higher risk of developing papules occurs in long-distance runners who are overweight and perform rapid starting and stopping motions.	[1,2,4-6]
Runner’s purpura	This is exercise-induced purpura, which can present on the lower legs as painful, paresthetic, or pruritic erythematous urticarial or purpuric plaques. Leukocytoclastic vasculitis is demonstrated on biopsy. It may be noted in runners who vigorously exercise in hot weather conditions on their legs and face.	[1,2]
Runner’s rump	Exercise-induced purpura presents as hyperpigmentation on the superior portion of the gluteal cleft of long-distance runners from the small ecchymoses that may occur at this location. Wearing a fanny pack may also predispose the runner to this condition.	[1,2,6]
Sports-associated clothing related axillary tangled clumped hairs (SCRATCH)	This is a unique form of ambulatory (running or walking) alopecia that is related to the clothing that the runner is wearing. Specifically, repetitive movement of the arms results in the seams of the shirt rubbing against the person’s axillary hairs; the hairs become painfully tangled and clumped. Once the shirt is removed, the tangled and clumped hairs need to be removed; if the hairs are forcefully detached from the skin, localized areas of temporary alopecia develop.	Current report
Talon noir	This is also known as black heel or calcaneal petechiae. It results from bleeding into the epidermis and eventually into the stratum corneum and presents as discrete brown or blue-black macules.	[1,2,4,6]
Transient plantar urticaria	This is the development of tender macules and papules on the feet. Neutrophils around dermal blood vessels are observed on biopsy. The condition is rarely diagnosed, and its pathogenesis remains to be determined.	[2]

In addition, various miscellaneous conditions have been described in reports of individual runners or multiple athletes who participate in ambulatory activities including not only running but also hiking and jogging and walking (Table 6) [1-3,16-20]. 

**Table 6:** Miscellaneous conditions associated with running IgG, immunoglobulin G

Condition	Comment	References
Aplastic anemia	A 26-year-old male marathon runner used rubber cement on his foot blisters to attach adhesive tape each day during two- to three-hour runs and marathons. After a year, he experienced decreased exercise tolerance, petechiae, spontaneous bleeding, and episodic hematochezia and melena. Laboratory studies demonstrated pancytopenia (hemoglobin, 10.8 g/dL; leukocytes, 3,300/cu mm; and platelets 18,000/cu mm). The bone marrow aspirate and biopsy showed a very hypocellular marrow. He did not improve after four months of oxymetholone (150 mg/day). His aplastic anemia was attributed to the benzene in the rubber cement.	[16]
Flushing	After a strenuous workout, some athletes develop flushing. The flushing may be limited by the lines of demarcation (Futcher’s lines)	[2]
Linear focal elastosis	A 16-year-old girl presented with horizontal bands that were only present both above and below each knee of three years duration; she had been training and running marathons since age 13. Biopsy showed increased elastin fibers in the dermis; the Verhoff Van-Gieson stain showed that the increased fibers were thin, wavy, elongated and fragmented. A diagnosis of linear focal elastosis was established.	[17]
Post ambulatory swollen hands (POTASH)	This is an idiopathic, usually asymptomatic, condition, albeit not uncommon, that occurs in individuals who participate in activities that involve ambulation (such as hiking, jogging, running, and walking). It is not dependent on the ambient temperature and may begin after approximately an hour of ambulating. The hands progressively become markedly enlarged and swollen; the person is not able to clench their hands into a tight fist. Typically, within two or fewer hours after the person stops ambulating, the swollen hands spontaneously return to their normal presentation, and the person can make a tight fist.	[18]
Reactive angioendotheliomatosis	A previously healthy 65-year-old male marathon runner presented with a 12-month history of intermittent painful, symmetrically distributed erythematous plaques on the ears (greater helices), flanks, and legs; some of the lesions ulcerated and became necrotic. The lesions were preceded by pruritus and were worse in cold weather; they improved in warm weather and when he wore protective clothing. Biopsy showed a benign appearing vascular proliferation in both the dermis and subcutaneous fat; the vessels contained organized thrombi. A diagnosis of reactive angioendotheliomatosis was made. Laboratory evaluation demonstrated serum cryoglobulins (monoclonal IgG kappa), free kappa light chains (on serum electrophoresis and in the urine), and 12 percent clonal plasma cells (on bone marrow biopsy); a diagnosis of type I cryoglobulinemia was made. Treatment with dexamethasone and thalidomide was started; the latter caused bradycardia and was switched to lenalidomide. All skin lesions and lab abnormalities completely resolved; treatment was stopped after 18 months, and follow-up at two years showed no recurrences. He continued to run marathons even in cold weather.	[19]
Solar purpura	This occurs in skin that has either age, actinic damage, or corticosteroids have altered the surrounding connective tissue support of the cutaneous blood vessels.	[1]
Subcutaneous edema	In a study of runners of an ultramarathon (250 kilometers over six days), ankle and foot swelling attributed to edema in the subcutaneous tissues was observed. Over the course of the race, the edema increased. The edema was observed not only in the lower limbs but also in the hands. This may be the same condition as POTASH.	[3]
Tolio	This is a catch-all term to describe the annoying and painful cutaneous foot experienced by unprepared river runners and hikers. It is speculated that the prolonged exposure to cold water causes the foot rashes; in addition to the environmental aquatic exposure, the rash is also partially attributed to the footwear. The foot maladies have also been referred to as boatman’s foot, canyon foot rot, holio, and river rot. Several conditions are part of tolio; they include bacterial superinfection (of the underlying skin disorders), chilblains (perniosis), irritant dermatitis, onychomycosis, pitted keratolysis, and tinea pedis.	[20]

Hair-associated changes from sports have previously been described; they tend to occur because of a localized condition. Dermatophyte infection and friction-related tractional alopecia have been described. The man in this report experienced localized temporary alopecia after his tangled and clumped axillary hair was manually removed; follow-up examination, one month later, demonstrated early regrowth of the hair in the areas of hair loss.

Localized alopecia can result from fungal infection or other etiologies. It has been described from dermatophyte infection in non-runners, such as tinea capitis and tinea corporis in wrestlers [5]. Potentially, tinea corporis could develop beneath the tight-fitting occlusive clothing that is worn by runners on their abdomen, legs, and torso.

In addition, localized frictional alopecia can result in hair loss occurring at the site of the exogenous source of irritation or traction. In 1984, a young woman was reported with acquired linear transverse scalp hair loss. She would wear a tight-banded, wide-stripped heavy headphone on the top of her head when she would jog daily. The hair loss stopped when she switched to a lighter headpiece. Dr. Copperman suggested that the woman’s condition be referred to as “alopecia walkmania” [12]. Subsequently, in 2006, Drs. Mailler-Savage and Adams recommended that the woman’s traction-related hair loss be designated as “jogger’s alopecia” [1].

Frictional alopecia of the distal legs is a tractional alopecia localized to the lower extremity; it is located below the knee in a distribution that corresponds to individual’s socks or footwear or both. A middle-aged man began a part-time occupation as a soccer referee; as part of his required uniform, he had to wear tight-fitting socks that extended up his legs higher than his calves. His new job as a soccer referee required him to be continuously running up and down the soccer field, two or three nights each week. Within three months after the soccer season began, he noticed that the hair on his legs had disappeared; the distribution of the alopecia corresponded to the location of the socks on his legs and was attributed to the friction caused by the socks continually rubbing against his skin during the soccer games [14].

Tangled and clumped axillary hair is related to the clothing that a walker or runner is wearing. SCRATCH is a unique sports-associated condition. Although SCRATCH may be a common condition, I am not aware of any prior case reports of affected individuals. To the best of the author's knowledge, SCRATCH has not been described in the medical literature; in May 2025, a search using PubMed did not reveal any related publications when the following terms were evaluated (either alone or in combination): alopecia, athlete, axilla, axillary, clumped, hair, hair loss, scratch, sports, and tangled. 

In addition to SCRATCH, clothing has been related to other sports-associated dermatologic conditions in runners such as chafing and jogger’s nipples [1,2,4-6]. Like SCRATCH, clothing has resulted in hair loss in individuals with frictional alopecia of the distal legs; the tractional alopecia that occurred was associated with the socks that they were wearing [14].

Clothing-related dermatological problems in runners have also previously been observed by a medical team of 57 people who were evaluating the runners of the 29th Sultan Marathon des Sables that took place in Morocco on April 4, 2014, to April 14, 2014. The endurance race of approximately 250 kilometers took place over six days in five self-contained stages in the desert. Repetitive friction of the clothing rubbing the skin not only resulted in chafing but also contact dermatitis caused by the chemical additives in the running apparel [3].

SCRATCH is symptomatic during the running event. The man from this report experienced pain when he moved his arms. After the race has been finished, the pain persisted while the shirt remained in contact with the body. Even after removal of the shirt, movement of the arm stretched the skin beneath the tangled and clumped axillary hairs, resulting in pain. The painful symptom completely resolved after the affected hairs were removed.

The diagnosis of SCRATCH involves correlation between the athlete’s activities, the presenting symptoms, and the appearance of the affected hairs. The athlete must be participating in an ambulatory sport (such walking or running), wearing a shirt with seams that contact the axillae, and repetitively moving the arms forward and back either adjacent to the flank or in front of the chest. Pain eventually develops when the tangled or clumped hairs are stretched during arm movements.

Examination of the axillae confirms the diagnosis. Both clumped matted masses of hair and linearly arranged tangled hairs are present among the normal-appearing axillary hairs. Based on the clinical scenario, the symptoms, and the morphology of the altered hairs, the diagnosis of SCRATCH is established; there are no other diagnostic possibilities when all the components of SCRATCH are present.

Treatment requires removal of the affected hairs; in the reported patient, if he moved his arms, the maximum pain ranged from two to four, on a scale from zero (painless) to 10 (most severe). Manual traction was applied to remove the hair masses from the body of the man in this report. A more compassionate manner of therapy would be to use a scissor to cut the tangled and clumped hair as close to the base of the hair and gently detach them.

The pathogenesis of SCRATCH is related to the repetitive rubbing contact of the clothing against the axillary hairs. The athlete in this report was wearing a 100 percent cotton shirt with prominent seams in the axillary region. He had been walking for about three hours before symptoms developed.

The mechanism for the alteration of the hair is attributed to the interaction between the seams of the clothing and the loose axillary hair. However, no clothing was present in either the masses of hair clumps or the linear tangled hair; in addition, hair was not found to be caught in the fibers of the shirt seams. Hence, the material from the shirt became the source of irritation that resulted in the abnormal hair formation.

The incidence of SCRATCH remains to be determined. SCRATCH does not occur in individuals who keep their axillary hair short by frequently shaving the area. Based on the observed characteristics of SCRATCH, it is reasonable to postulate that a similar condition might be observed in the inguinal region of athletes.

Prevention of SCRATCH can easily be achieved by shaving the axillary hair prior to running events. However, if the shaving is only performed periodically, there is a potential to develop pseudofolliculitis when the hair regrows. Alternatively, in contrast to shaving the axillary hair, the hairs can be significantly shortened by cutting them with a scissor which would prevent the subsequent possibility of pseudofolliculitis.

Another preventative strategy would be wearing clothing that does not occlude the axillae, such as a tank top shirt. However, these shirts do not provide the same amount of warmth to the participant. It is reasonable to postulate that applying a salve to the axillary hairs might be an effective intervention to prevent the condition; hence, the axillary hairs would adhere to the body and not rub against the shirt.

There are some limitations of this report. The report only describes a single patient with SCRATCH. Additional descriptions of walkers and runners with this condition should be published in the medical literature as individual case reports and case series.

Another limitation of this report was that the short follow-up on the reported patient. However, during the subsequent month, hairs in the sites of alopecia began to grow. Ideally, a longer period of follow-up would be beneficial.

Future research and surveillance to better establish the incidence and prevention strategies of SCRATCH would contribute to better characterizing these salient features of the condition. Studies should be performed to establish the incidence of SCRATCH in half marathon and full marathon participants. Evaluation of additional patients with SCRATCH might provide additional insight into methods that can be incorporated to prevent its development.

## Conclusions

Athletic participants can develop various conditions that affect their skin, mucosa, hair, and nails. Sports-related disorders occur in runners. A unique cutaneous condition affecting the axillary hairs of a 66-year-old man developed while he was participating in a half marathon. His axillary hairs became painfully tangled and clumped; this resulted from the repetitive friction and rubbing to his arm pits from the shirt he was wearing. Temporary localized patches of alopecia occurred when the affected hairs were removed. SCRATCH is the acronym that has been designated to describe this condition: sports-associated clothing related axillary tangled clumped hairs. SCRATCH results from the seams of the shirt rubbing against the axillary hairs and creating a mass of tangled and clumped hairs that are painful when the runner moves their arms. Focal areas of alopecia in the affected axillae may result from the removal of the hair masses; spontaneous resolution of the tractional alopecia resulting from the hair removal eventually occurred in the reported runner. The incidence of SCRATCH remains to be established; the condition only occurs in individuals who do not regularly shave their axillary hairs. Shaving the axillary hairs prior to running, applying a salve to the axillary hairs so that they do not adhere to the shirt, or wearing shirts that do not rub against the axillae can prevent SCRATCH. In conclusion, SCRATCH can be added to the list of potential dermatologic conditions associated with sports that can occur in runners.

## References

[1] Mailler-Savage EA, Adams BB (2006). Skin manifestations of running. J Am Acad Dermatol.

[2] Helm MF, N Helm T, F Bergfeld W (2012). Skin problems in the long-distance runner 2500 years after the Battle of Marathon. Int J Dermatol.

[3] Descamps V, Claessens YE, Doumenc B (2017). Skin manifestations in ultramarathon runners: experience in the Marathon des Sables 2014. Br J Dermatol.

[4] Purim KS, Leite N (2014). Sports-related dermatoses among road runners in Southern Brazil. An Bras Dermatol.

[5] Adams BB (2002). Dermatologic disorders of the athlete. Sports Med.

[6] Carr PC, Cropley TG (2019). Sports dermatology: skin disease in athletes. Clin Sports Med.

[7] Houston SD, Knox JM (1977). Skin problems related to sports and recreational activities. Cutis.

[8] Forouzan P, Cohen PR (2020). Erythrmasma revisited: diagnosis, differential diagnoses, and comprehensive review of treatment. Cureus.

[9] Mobacken H, Nordin P (1987). Molluscum contagiosum among cross-country runners. J Am Acad Dermatol.

[10] Greywal T, Cohen PR (2015). Pitted keratolysis: successful management with mupirocin 2% ointment monotherapy. Dermatol Online J.

[11] Ambros-Rudolph CM, Hofmann-Wellenhof R, Richtig E, Müller-Fürstner M, Soyer HP, Kerl H (2006). Malignant melanoma in marathon runners. Arch Dermatol.

[12] Copperman SM (1984). Two new causes of alopecia. JAMA.

[13] Cohen PR, Eliezri YD, Silvers DN (1991). Athlete’s nodules. J Am Acad Dermatol.

[14] Zhao J, Cohen PR (2016). Frictional alopecia of the distal legs: case series and review. Dermatol Online J.

[15] Cohen PR, Schulze KE, Nelson BR (2007). Subungual hematoma. Dermatol Nurs.

[16] Roodman GD, Reese EP Jr, Cardamone JM (1980). Aplastic anemia associated with rubber cement used by a marathon runner. Arch Intern Med.

[17] Kaur I, Jakhar D, Bhattacharya SN, Sharma S (2019). Linear focal elastosis localized to bilateral knee of a marathon runner. J Postgrad Med.

[18] Cohen PR (2024). Post ambulatory swollen hands (POTASH): a case report. AME Case Rep.

[19] Boyapati A, Khan S, Mar A, Sheridan A (2013). Reactive angioendotheliomatosis associated with cryoglobulinemia in a marathon runner. Dermatol Online J.

[20] Myers TM, Bigler CJ, Maurer MB, Gaither ME, Taylor WM (2020). Tolio: foot rot in Grand Canyon river runners. Wilderness Environ Med.

